# Immune Checkpoint Inhibitor-Induced Myositis Myocarditis and Myasthenia Gravis (MMM) Overlap Syndrome in a Rural District General Hospital: An Institutional Case Series Involving Four Patients

**DOI:** 10.7759/cureus.90106

**Published:** 2025-08-14

**Authors:** Umar Ismail, Elisabeta E Both, Helen Brown

**Affiliations:** 1 Internal Medicine, Withybush General Hospital, Hywel Dda University Health Board, Haverfordwest, GBR; 2 Acute Oncology, Hywel Dda University Health Board, Haverfordwest, GBR

**Keywords:** cancer immunotherapy, emergency oncology, ici, immune-related adverse event (irae), immuno-oncology, ipilimumab nivolumab, myasthenia gravis (mg), myocarditis, myositis, pembrolizumab toxicity

## Abstract

Immune checkpoint inhibitors (ICIs) are commonly used for the treatment of some advanced cancers. Although effective, they can cause side effects. This case series describes four patients treated for ICI-induced myositis, myocarditis and myasthenia gravis (MMM) overlap syndrome at a rural general hospital in the United Kingdom between 2023 and 2025. To the best of our knowledge, this is the first open-access case series describing its management specifically in a rural, low-resource setting. Patients were exclusively male with a mean age of 68.9 years and had received either dual nivolumab/ipilimumab or pembrolizumab monotherapy for metastatic melanomas, renal cell carcinoma or synchronous oesophageal and lung cancer. Symptoms included fatigue, proximal muscle weakness, dysphagia, diplopia, and dyspnea. High-dose intravenous methylprednisolone was the mainstay of treatment, with additional immunomodulators like mycophenolate mofetil, tocilizumab, tacrolimus and intravenous immunoglobulins added based on clinical progression and specialist input. Two patients recovered with treatment and remained progression free following ICI discontinuation, while two patients died due to respiratory failure, likely related to delayed initiation of myasthenia gravis-directed therapy. Prominent bulbar symptoms at presentation and high myasthenia gravis composite scores appear to correlate with poor outcomes. Early access to diagnostics such as autoantibodies for MG, tissue biopsy and electrodiagnostics was often limited by logistical barriers. However, biomarkers like creatine kinase, troponins and imaging modalities were consistently available. This series highlights the challenges of managing MMM syndrome in non-specialist resource-limited settings and the potential dangers of delayed immunosuppression. A high index of suspicion, early multidisciplinary involvement, and rapid initiation of therapies are critical to improving outcomes. Furthermore, this case series supports the need for simplified, context-appropriate guidelines that prioritise clinically impactful investigations and also highlights the emerging potential of the neutrophil-to-lymphocyte ratio as a practical prognostic tool. As ICI indications expand, awareness of MMM overlap syndrome among frontline clinicians is essential to reduce treatment delays and prevent avoidable mortality.

## Introduction

Cancer cells express tumour-associated antigens which are recognised as “foreign” by the immune system, resulting in T-cell activation through a tightly regulated process of cellular antigen presentation [1]. However, tumours develop defences to escape this process of immune recognition, including the delivery of inhibitory signals to immune cells [1]. This pathway is targeted by immune checkpoint inhibitors (ICIs), which are monoclonal antibodies. Since gaining approval for use from the United States Food and Drug Administration in 2011, ICIs have become an integral part of clinical guidelines for the management of some metastatic and non-metastatic cancers, representing a paradigm shift in solid tumour oncology with improved prognosis for many patients [2]. Their mechanism of action involves inhibiting cytotoxic T lymphocyte-associated antigen 4 (CTLA4) and programmed cell death 1 (PD-1) on T-cells, or its ligand (PDL-1), leading to enhanced T-cell activation and immune activity against cancer cells. Newer agents like relatlimab, which target lymphocyte activating gene 3 (LAG-3), are also gaining traction to address the low response rate noticed with some established ICIs [3].

Despite showing impressive anti-tumour efficacy and better safety profile compared to conventional chemotherapy [4], ICIs are associated with wide-ranging toxicities termed immune-related adverse events (irAEs) which affect multiple organs [5]. Inflammation affecting skeletal muscles (myositis), cardiac muscles (myocarditis) or the neuro-muscular junctions (MG) can occur alone or in various combinations. The reported incidences of myositis, myocarditis and myasthenia gravis in all ICI-treated patients are 1%, <5% and 0.1-0.2% respectively [6]. With a reported incidence below 1% among ICI-treated patients [5,6], a particularly lethal combination of irAEs is the myositis, myocarditis, and myasthenia gravis overlap, referred to as MMM overlap syndrome. The simultaneous occurrence of these three conditions presents both a diagnostic and management challenge with a correspondingly high morbidity and mortality [5]. Given the rarity of this syndrome, its clinical features, pathophysiology, prognostic indicators, and outcomes remain not well understood by the non-specialist physician. The incidence of irAEs, including potentially devastating ones like MMM overlap, is expected to rise as ICI use expands globally.

Although the underlying mechanisms for the overlapping tissue inflammation seen in some ICI-treated patients remain unclear, molecular mimicry (potentially shared epitopes between the tumour and myocardium) and the critical role of PD-1 signalling pathways in regulating immune responses in these tissues may be responsible [5,7]. Other considerations include the release of intracellular antigens upon tumour necrosis, T-cell infiltration due to overexpression of PD1 on cardiomyocytes, or the release of proinflammatory cytokines [8]. The process appears to be mainly cell-driven, as most respond well to treatments directed against T-cells, and a large proportion do not test positive for autoantibodies typically seen in similar primary autoimmune processes. However, arguments have been made in support of the role of humoral immunity as patients appear to respond to therapies directed against this aspect of the immune response (e.g., intravenous immunoglobulin and plasma exchange). Complement deposition within capillaries (pericapillary C4d) was reported in two cases of ICI-induced myocarditis, suggesting a component of antibody-mediated injury [5]. In this case series, we give an account of the management of MMM overlap syndrome in our facility and explore the unique challenges of managing such a complex condition in a non-specialist rural hospital. All consecutive patients diagnosed with MMM overlap syndrome between 2023 and May 2025 were included.

While published reports from the United Kingdom, United States, Canada, Australia, and Europe have provided valuable insights into the pathophysiology and management of this syndrome, they are almost exclusively from tertiary, urban, or academic medical centres with access to advanced critical care and specialist diagnostics. To date, only isolated open-access reports, including our prior publication, have described the syndrome in rural or low-resource healthcare environments. To the best of our knowledge, this is the first open-access case series documenting the recognition and management of ICI-induced MMM syndrome in a rural, low-resource UK district general hospital, offering unique insights into the diagnostic, therapeutic, and system-level challenges in such settings.

## Case presentation

We conducted a retrospective review of all adult patients diagnosed with ICI-induced MMM overlap syndrome at our hospital between January 2023 and May 2025. Eligible patients were identified through multidisciplinary team (MDT) discussions, electronic records, and hospital admission logs. Clinical data, laboratory results, imaging findings, and treatment details were extracted from electronic medical records by the treating clinicians.

Diagnostic confirmation of MMM syndrome was based on the presence of compatible clinical features (e.g., proximal muscle weakness, ptosis, dyspnoea), supportive biomarkers (e.g., elevated creatine kinase, troponin), and cardiac or neuromuscular imaging, consistent with current guideline definitions, recent literature and consultation with experts. Where available, myasthenia gravis composite (MGC scores, neutrophil-to-lymphocyte ratios (NLR), and CTCAE grading were recorded. All patients received ICI therapy within four weeks of symptom onset. 

Case 1

A 67-year-old man with unresectable melanoma of the neck and lymph node metastasis on nivolumab and ipilimumab combination therapy presented 1 week after the second cycle of immunotherapy with shortness of breath, generalised muscle aches, weakness and difficulty walking. No prior history of autoimmune disease was known at the time of presentation. He was independent and self-caring with a WHO performance score of 1 at the time of assessment. The patient was tachypneic and hypoxic at the time of admission, requiring 4 litres of oxygen via nasal cannula to maintain oxygen saturation above 94%. Proximal muscle weakness was noted on examination with reduced lower limb deep tendon reflexes; otherwise, no other neurology was identified (swallowing was fine; no ptosis). CK was elevated at 10866U/L while Troponin T and I were 498ng/L and 310ng/L, respectively (Table 1). Arterial blood gas showed type 1 respiratory failure (T1RF). MGC score was 9, forced vital capacity (FVC) was 32% of predicted, while electrocardiograph (ECG) and echocardiograph (ECHO) showed sinus rhythm and normal left ventricular function, respectively. Magnetic resonance imaging (MRI) of the spine and brain excluded metastatic disease.

**Table 1:** Admitting laboratory results CRP: C-reactive protein; AST: Aspartate transaminase; ALT: Alanine transaminase; Trop: Troponin; NTProBNP: N-terminal pro brain natriuretic peptide; NLR: Neutrophil-to-lymphocyte ratio.

Labs	Value	Reference range
Metabolic panel		
Urea	4.3 mmol/L	2.5-7.8 mmol/L
Creatinine	88 mmol/L	58-110 mmol/L
Potassium	4 mmol/L	3.5-5.4 mmol/L
Sodium	139 mmol/L	135-145 mmol/L
Calcium	2.4 mmol/L	2.2-2.6 mmol/L
Cortisol	520 mmol/L	>420 mmol/L
CRP	15 mmol/L	< 5.0 mmol/L
Albumin	43 g/l	35-55 g/L
AST	553 U/L	< 50 U/L
ALT	469 U/L	<50U/L
Hepatitis panel	negative	NA
Liver autoantibodies	negative	NA
Cardiac and musculoskeletal labs		
CK	10389 U/L	40-320 U/L
Trop T	393 ng/L	< 14
Trop I	310ng/L	< 14
NTProBnP	88 pg/L	< 125pg/L
Blood cell counts		
Lymphocytes	0.9	1.0-4.0 ku/L
Neutrophils	7.9	1.8-7.5ku/L
NLR	8.78	NA

**Figure 1:**
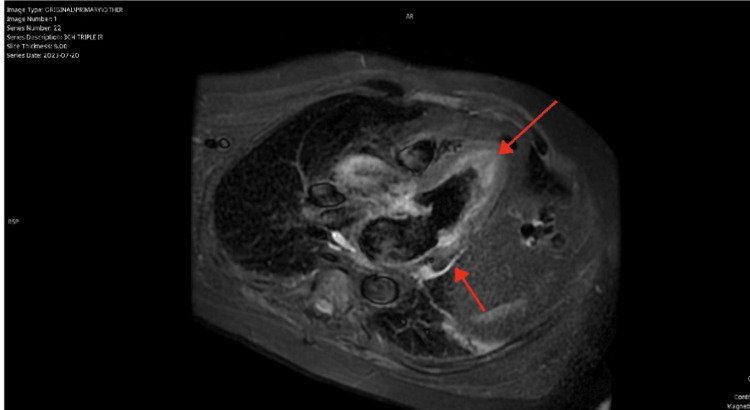
Cardiac MRI (3 chamber triple IR) showing increased myocardial signal intensities in the basal inferolateral and mid-cavity anterolateral segments in keeping with myocarditis (red arrows) IR: Inversion recovery; MRI: Magnetic resonance imaging

**Figure 2:**
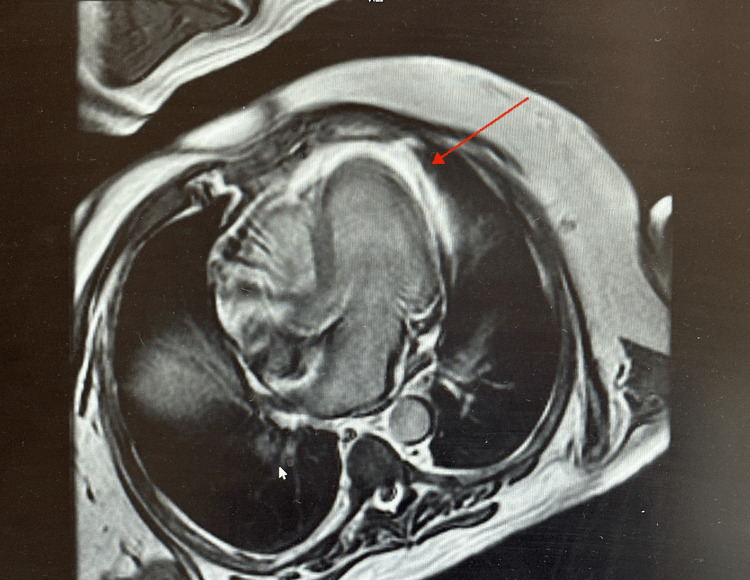
Cardiac MRI (T2 weighted STIR) showing increased signal intensities in the pericardium due to pericardial effusion (red arrow) which in the context of myocarditis suggests pericardial inflammation STIR: Short tau inversion recovery

The patient was treated with Intravenous (IV) methylprednisolone 2mg/kg/day initially, with daily FVC monitoring and neurological examination. Steroid dose was considered inadequate and was corrected to the guideline-recommended 1000mg/day by day 6 of admission after cardiology review. Although cardiac MRI was done and showed changes consistent with myopericarditis (Figures 1, 2), MRI of the musculoskeletal system was not pursued as it was felt that it wouldn’t change management. Muscle biopsy and electrodiagnostic tests (electromyography, nerve conduction studies) were not considered during the admission due to logistical reasons; this doesn’t seem to have impacted clinical decision making. Neuromuscular autoantibody screen (Anti-AchR) was negative. Although transaminases were severely elevated, liver autoantibodies and infectious hepatitis screen were negative as shown in Table 1, suggesting that the transaminases were possibly raised due to muscle inflammation. Mycophenolate mofetil (MMF) 500mg twice daily was added on the advice of an immune-oncology specialist due to concerns about slow improvement in symptoms even after escalation of the steroid dose. The patient steadily improved after this and was successfully weaned off oxygen by day 12 of admission. FVC improved to 45%.

Although the patient avoided intensive care admission and intubation, the clinical course was marked by slow improvement in both symptoms and biomarkers (CK and Troponins). The patient was eventually discharged on oral prednisolone and MMF by day 15 of admission. NLR fell to 3.75 from 8.78 at admission. ICIs were permanently discontinued as both myositis and myocarditis were judged to be grade 4 (life-threatening) per the common terminology criteria for adverse events (CTCAE). Weekly outpatient monitoring with CK, Troponins (T; I) and ECG was recommended. MMF was discontinued early after discharge due to sudden derangement of liver function, suggesting MMF-related liver toxicity. The patient did well on follow-up and was successfully weaned off steroids. Patient's disease remained stable with the patient remaining progression free one year after discharge.

Case 2

An 84-year-old man with metastatic melanoma of the back and right groin nodal disease with a previous remote history of prostate cancer (under surveillance after full response to treatment) presented two weeks after the first cycle of adjuvant pembrolizumab with severe fatigue, weakness, shortness of breath and double vision. The patient denied any swallowing difficulty or muscle aches. No previous history of autoimmune disease was known prior to commencement of immunotherapy. The WHO performance score was 2 at the time of presentation, and the patient was living independently. The patient was enrolled in the PROPHETIC trial prior to this admission. Patient's symptoms had been ongoing for four days prior to admission, but the patient declined hospital review early on. 

On examination, the patient was vitally stable at the time of admission. Proximal myopathy, left eye ptosis, ophthalmoplegia and diplopia were noted, FVC was 38% of predicted, and MGC score was 19. ECG showed sinus rhythm with right bundle branch block (RBBB). Admitting CK, Troponin T, and I were 5346U/L, 4184ng/L and 11066ng/L, respectively, as shown in Table 2. Although the MRI of the brain returned normal, the whole spine MRI showed some bone metastasis. The patient was initially treated with oral prednisolone at 0.5mg/kg/day; there was no concern about MMM syndrome at this point. Despite high CK and troponin, the admitting team suspected MG related to immunotherapy. The patient's oncologist was contacted, who suggested following the European Society of Medical Oncology (ESMO) guidance for MG management and highlighted the possible myositis.

**Table 2:** Admitting laboratory tests CRP: C-reactive protein; AST: Aspartate transaminase; ALT: Alanine transaminase; Trop: troponin; NTProBNP: N-terminal pro brain natriuretic peptide; NLR: Neutrophil-to-lymphocyte ratio; Anti-AchR: Anti-Acetylcholine receptor antibodies.

Labs	Value	Reference range
Metabolic panel		
Urea	7.8mmol/L	2.5-7.8 mmol/L
Creatinine	100mmol/L	58-110 mmol/L
Potassium	4.4mmol/L	3.5-5.4 mmol/L
Sodium	138mmol/L	135-145 mmol/L
Calcium	2.64mmol/L	2.2-2.6 mmol/L
CRP	19 mmol/L	< 5.0 mmol/L
Albumin	35g/l	35-50 g/L
AST	391U/L	< 50 U/L
ALT	246 U/L	< 50U/L
Coagulation profile	normal	NA
Cardiac and MSK labs		
CK	5346U/L	40-320 U/L
Trop T	4184ng/L	< 14
Trop I	11066ng/L	< 14
NTProBnP	4127pg/L	< 125pg/L
Anti-AchR	negative	NA
Blood cell counts		
Lymphocytes	0.4	1.0-4.0 k/uL
Neutrophil	7.3	1.8-7.5 ku/L
NLR	18.25	NA

IV methylprednisolone 1000mg/day was recommended and commenced after neurology discussion on the second day of admission due to concerns about concurrent myositis. The patient clinically deteriorated by day 3 with more weakness and fatigue, so IV immunoglobulin (IVIG ) was commenced and was escalated to HDU due to respiratory failure. At this point, ECHO showed left ventricular ejection fraction (EF) of 35% and a cardiology review was requested. MRI of the quadriceps and myocardium were booked but later abandoned as the patient became too unwell. Neuromuscular autoantibody screen (anti-AchR) was negative. Plasma exchange (PLEX) was considered on day 8 of admission, although this was abandoned due to concerns about heart failure. 

FVC deteriorated to 20% by day 9, and MMF was added after discussion with the oncologist. Guideline-directed heart failure management was advised after cardiology review. Additional immunosuppression with tocilizumab 8mg/kg/day, tacrolimus 3mg twice daily and pyridostigmine 30mg three times per day was added by day 10 on specialist advice in view of inadequate clinical response. Antibiotic prophylaxis with co-trimoxazole 480mg once daily was started given profound immunosuppression and infection risk. Respiratory function continued to deteriorate, and support was started with non-invasive ventilation (NIV/BiPAP). The patient was intubated by day 12 and transfer to the tertiary centre was considered again for PLEX, although later abandoned as the patient was deemed too unstable for transfer. Given the underlying metastatic cancer and age, only single organ support was agreed upon with intensivist. 

Of note, CK improved and normalised on treatment, Troponin T did improve but remained at a high level (800) while Trop I fell to 4000ng/L from the initial high of 11066ng/L suggesting deterioration was probably MG driven. Some clinical improvement was noted on day 13, so the patient was extubated. However, new atrial fibrillation and agitation were noted afterwards. Specialists agreed for further 48 hours of treatment, if no response, then to proceed with palliative care. The patient deteriorated rapidly and died on day 15 of admission. Toxicities (myositis, myocarditis and MG) were judged to be grade 5 (resulting in death) per CTCAE. Notably, despite other biomarker improvements seen above, NLR worsened from 18.25 at the time of admission to 41 on the last blood tests done prior to death. The NLR was 6.4 one month prior to this admission.

Case 3

A 68-year-old man with widely metastasised oesophageal adenocarcinoma and synchronous lung cancer developed progressive fatigue, ptosis, and slurring of speech four weeks after the fourth cycle of pembrolizumab. WHO performance score was 2 prior to this presentation. Interestingly, he had been diagnosed with grade 1 immunotherapy-related hepatitis just a week prior to the onset of these symptoms and was under surveillance with regular blood tests. Although he had some neuromuscular symptoms, they were believed to be at that point due to established frontal lobe metastatic disease. The patient was subsequently reviewed and commenced on oral prednisolone in the following week as transaminases continued to rise. Around this time, the patient's neuromuscular symptoms worsened, now having severe difficulty with neck control and worsening bulbar symptoms (difficulty with mastication, swallowing, and incoherent speech). Immunotherapy-related myasthenia gravis was suspected, and inpatient assessment was offered. Despite being made aware of the seriousness of these symptoms, the patient declined further assessment in the hospital at this time. The patient only agreed to hospital review and admission 11 days after the onset of symptoms following repeated persuasion by the oncology nurses as he felt his symptoms were not getting worse.

Although the patient had a three-litre oxygen requirement at the time of presentation to the hospital, he was otherwise vitally stable. General physical exam noted severe neck weakness with complete loss of neck control, proximal myopathy, quadriparesis, bilateral ptosis, diplopia, and slurring of speech. CK, troponin T and I were 685U/L, 707ng/L and 244ng/L, respectively, as shown in Table 3. NTproBNP was 1433 pg/L, and the NLR at the time of admission was 10.6; this was 6.67 four weeks prior. The MGC score was 14 and ECG and ECHO were normal. MRI brain confirmed stable previously known cerebral metastasis with no peri-lesional oedema or new lesions. He was treated with IV methylprednisolone with a very poor clinical response. Although his symptoms appeared to have been MG-driven, MG-directed therapies (IVIG, PLEX and pyridostigmine) were not commenced at the beginning of treatment; IVIG was started roughly 48 hours after admission. It is unclear whether this was to comply with guidelines, which suggest IV methylprednisolone as a first measure for MMM, with subsequent addition of other agents after reassessment in 72 hours [6]. The patient was suspected of having aspirated during a swallowing assessment. Nasogastric tube feeding and IV antibiotics were commenced. ABG showed type 2 respiratory failure for which the patient was commenced on non-invasive ventilation. Biologics (Infliximab; rituximab) and PLEX were considered by day 4 due to lack of response to steroids, but tacrolimus was favoured and commenced after discussion with the oncologist due to concerns about ongoing infection that may be worsened by biologics. Although other investigations (cardiac MRI, MRI of quadriceps, autoantibody screen) were planned, they were not completed as the patient rapidly deteriorated despite initial treatment. Logistical challenges would also have made some of the investigations difficult as they would have required coordination with the tertiary centre, and the patient was not stable for transport.

**Table 3:** Admitting laboratory tests CRP: C-reactive protein; AST: Aspartate transaminase; ALT: Alanine transaminase; Trop: troponin; NTProBNP: N-terminal pro brain natriuretic peptide; NLR: Neutrophil-to-lymphocyte ratio; NA: Not applicable

Labs	Value	Reference range
Metabolic panel		
Urea	7.0 mmol/L	2.5-7.8 mmol/L
Creatinine	71 mmol/L	58-110 mmol/L
Potassium	3.6 mmol/L	3.5-5.4 mmol/L
Sodium	135 mmol/L	135-145 mmol/L
Calcium	2.43 mmol/L	2.2-2.6 mmol/L
CRP	123 mmol/L	< 5.0 mmol/L
Albumin	37 g/l	35-50 g/L
AST	158 U/L	< 50 U/L
ALT	131 U/L	< 50U/L
Coagulation profile	normal	NA
Cardiac and Musculoskeletal labs		
CK	685 U/L	40-320 U/L
Trop T	707 ng/L	< 14
Trop I	244 ng/L	< 14
NTProBnP	1433 pg/L	< 125pg/L
Anti-AchR	Not done	NA
FBC		
Lymphocytes	0.5	1.0-4.0 ku/L
Neutrophil	5.3	1.8-7.5ku/L
NLR	10.6	NA

Discussions were had regarding transferring the patient to tertiary care where both neurology, cardiology and oncology services were available onsite, given his clinical complexity. Palliation was instead agreed after discussions between the family and the clinical team as prognosis was generally believed to be very poor. The patient died seven days after admission. IrAEs were judged to be grade 5 per CTCAE as death occurred. NLR at time of death was 33, almost a 3-fold increase compared to levels at time of hospital admission.

Case 4 

A 60-year-old man with a history of obesity, chronic kidney disease (CKD stage 1), atrial fibrillation, and clear cell renal cell carcinoma (RCC) with metastatic spread to the lungs post-nephrectomy presented to our emergency department with profound fatigue and shortness of breath two weeks after cycle 2 of nivolumab and ipilimumab. At the time of presentation, the patient was functionally independent with a WHO performance score of 1 and an International metastatic renal cell carcinoma database consortium (IMDC) score of 1. No underlying autoimmune disease was known at the time of presentation. 

Swallowing difficulty was also reported after repeated questioning. Notably, he denied chest pain or palpitations. No history of ptosis, diplopia or muscle pain was reported. Vital signs were stable at presentation to the emergency department. Neurological examination was unremarkable with full power in all limbs and normal reflexes; other systemic examinations were normal. The MGC score was 5 and FVC was 56% of predicted. Admitting labs (Table 4) demonstrated CK of 6,376 U/L, Troponin T of 1,694 ng/L and NTproBNP of 1369ug/L. Renal function was consistent with stable CKD stage 1 (patient's baseline) while liver function tests showed moderately raised transaminases (Table 4). Liver autoantibodies and coagulation profile were however normal. Full blood count was within normal limits and neuromuscular autoantibody screen (anti-AchR, antiMusK) was negative. NLR was 11.14 at the time of admission compared to 4.7 three weeks prior.

**Table 4:** Admitting laboratory results CRP: C-reactive protein,  AST: Aspartate transaminase,  ALT: Alanine transaminase, Trop: troponin,  NTProBNP: N-terminal pro brain natriuretic peptide,  NLR: Neutrophil to lymphocyte ratio, FBC: Full blood count, Anti-AchR: Anti-acetylcholine receptor antibodies, Anti-MusK: Anti-muscle-specific kinase antibodies

Labs	Value	Reference range
Metabolic panel		
Urea	10.8 mmol/L	2.5-7.8 mmol/L
Creatinine	115 mmol/L	58-110 mmol/L
Potassium	4.7 mmol/L	3.5-5.4 mmol/L
Sodium	135 mmol/L	135-145 mmol/L
Calcium	2.36 mmol/L	2.2-2.6 mmol/L
CRP	15 mmol/L	< 5.0 mmol/L
Albumin	41 g/l	35-50 g/L
AST	317U/L	< 50 U/L
ALT	321 U/L	< 50U/L
Cortisol	562 mmol/L	420 mmol/L
TSH	4.46	0.38-5.33mu/L
Free T4	17.4	8.0-18.0pmol/l
Coagulation profile	normal	
Cardiac and musculoskeletal labs		
CK	6376 U/L	40-320 U/L
Trop T	I694ng/L	< 14
Trop I	14ng/L	< 14
NTProBnP	1369 pg/L	< 125pg/L
Anti-AchR and Anti MusK	negative	NA
FBC		
Lymphocytes	0.7	1.0-4.0ku/l
Neutrophil	7.8	1.8-7.5ku/l
NLR	11.14	NA

**Figure 3:**
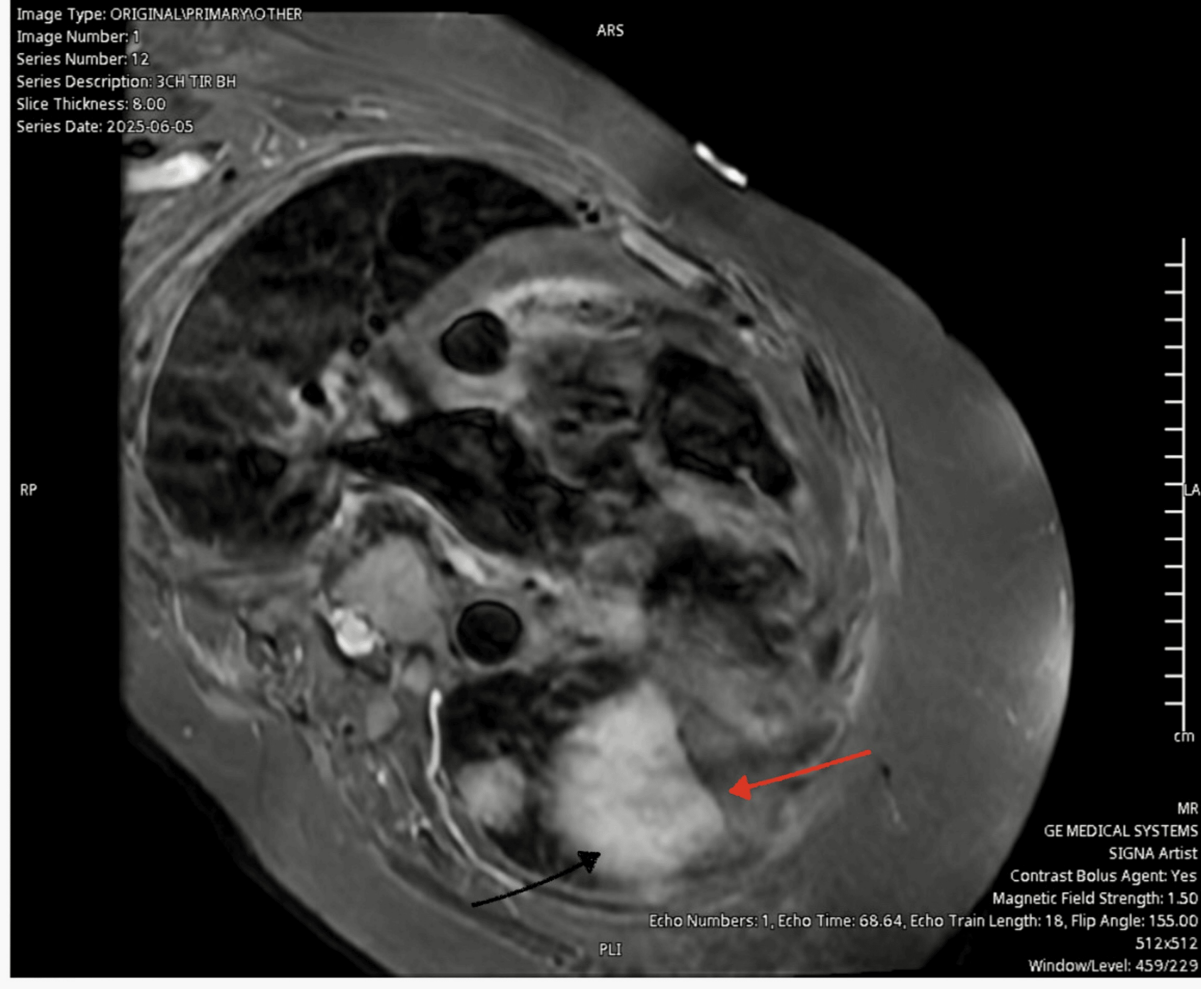
Cardiac MRI (3 chamber tau inversion recovery) showing increased signal intensities consistent with myocardial oedema and inflammation (red arrow)

**Figure 4:**
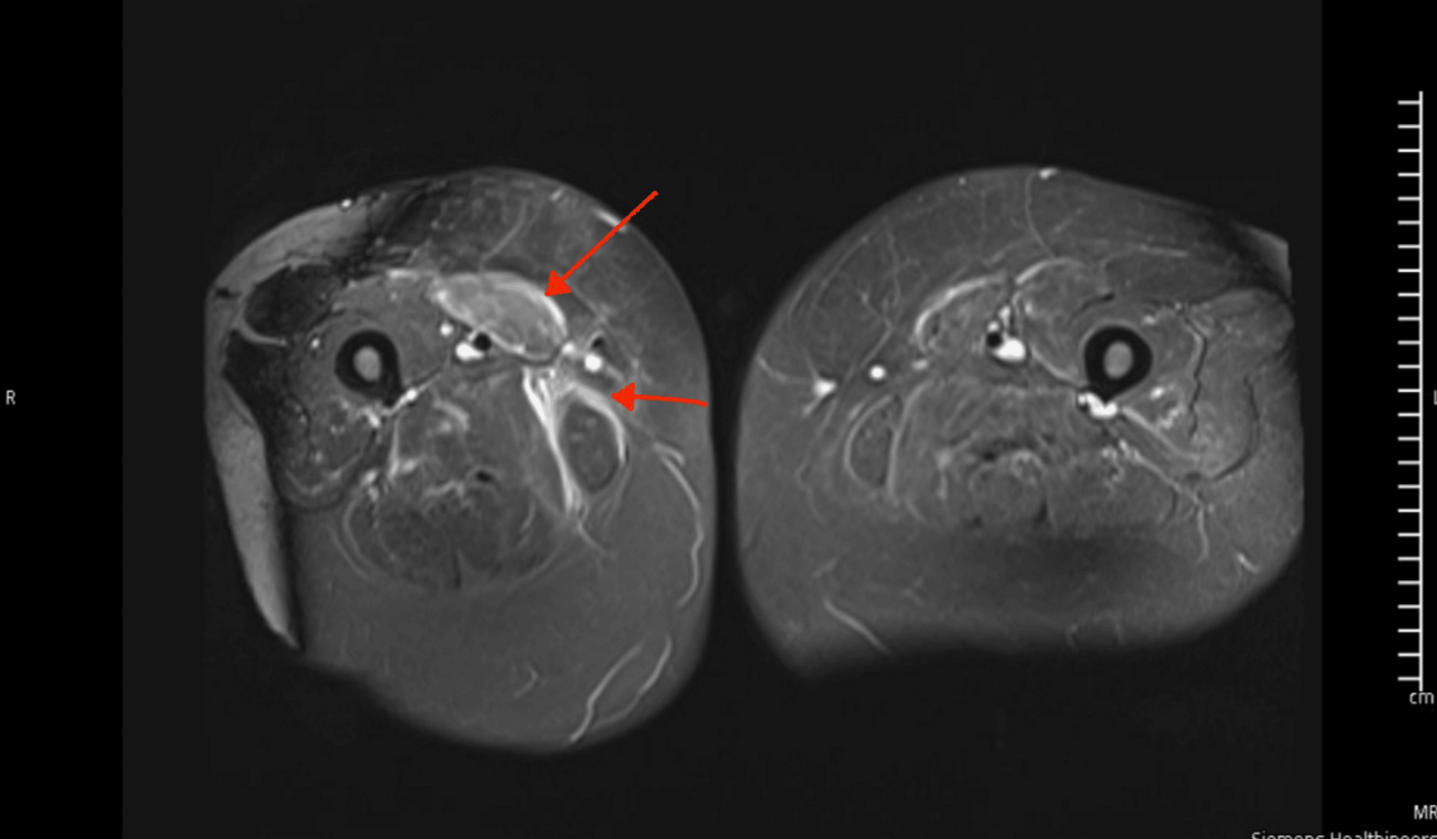
MRI of quadriceps (STIR) showing signal hyperintensities in keeping with muscle inflammation and oedema (myositis) STIR: Short tau inversion recovery

The admitting team initially commenced oral prednisolone 100mg; this was switched to IV methylprednisolone 1000mg/day less than 24 hours after admission. The cardiologist reviewed and commenced strict fluid input/output charting, low-dose bisoprolol to address mild tachycardia, IV furosemide and daily weights. The patient was moved to the coronary care unit for cardiac monitoring at the advice of the cardiologist. The patient was started on IVIG 2g/kg divided over five days, pyridostigmine 30mg daily, MMF initially 500mg twice daily and escalated to 1g twice daily after day three of treatment, and daily FVC monitoring and full neurological examination to assess for deterioration after further discussions with the oncologist. The patient showed marked clinical improvement by day 3 of immunosuppressive therapy. CK and troponinT levels decreased by more than 50% and swallowing normalised; the MGC score fell to zero. Although troponin I was requested early during the admission it was not processed initially because the lab staff didn't agree with the indication for testing. This was resolved after discussions, and it came back normal at 14ng/L 4 days into the admission, after the patient had already shown clinical improvement. Myocardial MRI was done two weeks after commencement of treatment and showed increased signal intensities on tau inversion recovery sequence in keeping with myocardial oedema and inflammation (Figure 3). MRI of the quadriceps obtained earlier during the admission showed diffusely increased signal intensities, most notable in the medial right thigh muscles in keeping with oedema and inflammation confirming myositis (Figure 4).

IV methylprednisolone was titrated down to 2mg/kg/day. Pyridostigmine was stopped by day 5 as all myasthenia symptoms resolved. The patient continued to show improvement and was switched to oral prednisolone by day 7 of admission; oral co-trimoxazole prophylaxis was commenced in view of immunosuppression. He was discharged on oral prednisolone and MMF with a weaning schedule and twice weekly outpatient blood tests and ECG monitoring by day 22. NLR was 18.6 at this point. MDT discussion was booked, and ICIs were stopped indefinitely as both myocarditis and myositis were judged to be grade 4 per CTCAE grading. Staging CT was organised to assess disease burden and plan next steps. The patient continued to show improvement after discharge and CK remained normal although troponin T remained elevated. He remained progression free on the CT scan done two months post discharge.

## Discussion

ICI-related toxicities like MMM overlap are rare, complex clinical syndromes, often with fulminant progression and prove challenging to manage. Mortality is very high (40-60 percent) even with aggressive treatment [5,9]. Navigating such a complex condition with a high likelihood of poor outcomes requires clinicians to have a comprehensive understanding of key risk factors, pathophysiology and likely symptoms at presentation in order to act quickly. Delay in instituting optimal management does affect outcomes. In our series, both patients 2 and 3 experienced substantial delays from diagnosis to starting treatment and had bad outcomes (death). Although other confounding factors like age, comorbidities, cancer type and staging likely influenced outcome, delay does appear to be a major feature in both their management.

Although small, our patient cohort shares similar demographic and oncological characteristics with those of MMM syndrome patients described in the literature. In their systematic review, Pathak et al. reported the median age of the patients at presentation as 71 years, with 67% of patients being male and 18% having metastatic melanomas. There was no consistent pattern of preexisting autoimmune disease, and the median number of doses until the development of the syndrome was just one dose [5]. Although the syndrome can develop in response to any type of immunotherapy, data suggest a higher likelihood with dual therapies (anti-PD-1/PDL-1 and anti-CTLA4 combinations) [7,10]. Similarly, our patient cohort was exclusively male, with an average age of 69.8 years and no underlying autoimmune disease before starting ICI therapy. Two patients (50%) had metastatic melanomas, one had RCC, and one notably had synchronous oesophageal and lung cancer. Two patients (50%) were on nivolumab and ipilimumab dual therapy, while two were on pembrolizumab monotherapy. All patients in our series experienced toxicity between cycles 1 and 4, with no consistent pattern in the timing of onset. Of note, higher MGC scores were noted in those on pembrolizumab monotherapy, who unfortunately all died. This observation that MG predominated in those on pembrolizumab monotherapy is consistent with pharmacovigilance data identifying pembrolizumab as carrying one of the highest risk signals for MG and MG crisis among ICIs. Additionally, retrospective reviews such as the one done by Safa et al. in 2019 showed that PD‑1 monotherapy accounts for nearly 90% of ICI-related MG cases [11], whereas CTLA‑4 inhibitors alone are less often implicated. Although combination therapy is associated with an accelerated and more severe presentation, this doesn't appear to be the case in our institution.

Fatigue (80%) and muscle weakness (78%) were the most common symptoms reported by patients in the referenced study above [5]. Our patients similarly reported these non-specific symptoms in addition to other specific ones. Earlier in this article, we noted that some patients may not recognise the seriousness of these symptoms because they occur commonly in cancer patients. Both healthcare providers and patients are likely to assume symptoms like new-onset fatigue to be due to the underlying cancer itself. This highlights the need for both better patient education and heightened awareness among oncologists, hospital doctors, and other multidisciplinary team members involved in the care of these patients in order to avoid fatal assumptions [7]. One of our patients was diagnosed with ICI-related hepatitis on the basis of raised transaminases, the source of which may have been muscle inflammation. It is noteworthy that hepatitis can occur concurrently with MMM overlap in rare cases, causing MMMH syndrome. An opportunity may have been missed at that point to check for other markers of myositis such as CK and aldolase, or explore symptoms of cardiac muscle inflammation. Myositis and MG were not suspected until a week later due to additional symptoms.

While current guidelines recommend confirmatory testing such as electromyography (EMG) and antibody panels to establish diagnosis, logistics can complicate management in low-resource settings. Our hospital lacks onsite autoantibody testing for MG, with turnaround times of up to four weeks. Electrodiagnostic tests and cardiac MRI must also be arranged in tertiary centres after both verbal discussion and emails with different teams. Notably, tests such as the MG autoantibody screen and EMG appear to have limited utility in clinical decision making and may be mostly academic. In clinical practice, treatment would proceed in much the same way regardless of whether some of these tests are positive or negative as seen earlier. It’s also useful to note that some of these autoantibodies are only positive in up to 66% of cases and lack sufficient sensitivity to rule out ICI-related MG [11]. In our series, the MGC score provided more timely and clinically relevant insight than autoantibodies. All patients tested for anti-AchR antibodies in our series (3 out of 4) were seronegative, consistent with prior reports that a significant proportion of ICI-induced MG cases are seronegative [5,11]. In contrast, MGC scores correlated with severity at presentation and predicted outcomes, with higher scores (≥13) seen in both patients who deteriorated and ultimately died. This suggests the need to further explore the role of bedside clinical scoring tools such as MGC score, particularly when access to confirmatory diagnostics is delayed or unavailable. More research is needed on what value autoantibodies and electrodiagnostic tests add. There is a need to streamline investigations to prioritise those that inform real-time clinical decision making and prognosis. More research is also needed to develop context-appropriate recommendations regarding diagnosis and management. 

Simple and readily available tests such as troponins, CK, NTproBNP, ECG and ECHO, alongside MRI and strategic application of clinical judgement appear to be sufficient to safely guide treatment escalation. Electrodiagnostic tests, tissue biopsy and autoantibodies have not played a significant role in the management of our patients and did not appear to have hampered decision making or produced any effect on outcomes. A case could be made for safely omitting them, especially in select contexts. They clearly don’t influence the choice of immunosuppressive agents or the decision to discontinue ICIs since the clinical presentation would be judged as a grade 3+ irAE per CTCAE regardless of whether myositis was biopsy-proven, or MG was confirmed with autoantibodies testing or electrodiagnostics.

While National and International treatment guidelines suggest starting with high-dose IV methylprednisolone (500-1000mg/day) and reviewing clinical and biomarker response prior to further escalation of treatment [6], in practice treatment is often individualised. There is often a choice to pursue early aggressive multi-pronged immunosuppression or follow the guideline-recommended approach. A case could be made for the former as the risk of deterioration is very high even in advanced centres with the best of resources. This approach aligns with the practice described in existing literature [5,12]. Head-to-head comparisons are needed to determine which approach is best. Consulting immunotherapy specialists early on is crucial, especially where there is limited local expertise. Notably, all four patients in this report received inappropriately lower doses of steroids at the beginning of treatment despite the widespread availability of guidelines through strategically placed QR codes in the hospital.

While immunosuppression may need to be sufficiently broad to target multiple stages of the immune response, especially in patients in whom steroid resistance is feared or suspected, the choice of immunosuppressives is often a balance between potential benefits and risk of toxicity (e.g. worsening heart failure, hepatotoxicity) and requires careful consideration. The options include cytokine inhibitors (anti-IL6, anti-TNF), conventional disease-modifying anti-rheumatic agents like MMF, CD20 inhibitors (rituximab) and other immunomodulators such as IVIG. One patient in our series (case 1) did experience MMF-induced hepatotoxicity soon after discharge, necessitating its discontinuation. Physicians may choose to start treatments like IVIG and MMF alongside steroids due to concerns about the risk of steroid resistance [13] or paradoxical worsening of myasthenia gravis in the context of high-dose steroids [11,14,15]. While this approach may be a deviation from guidelines, this may not only be reasonable in a life-threatening condition with a high likelihood of poor outcomes, but may also be supported by pathophysiologic reasoning. The role of steroids in controlling immune dysregulation in MMM syndrome might be limited by the constant presence of the original trigger; the circulating ICIs, as their half-life ranges from 14.7 to 27.3 days depending on the agent. Elimination of pathogenic antibodies and ICI monoclonal antibodies from the sera of patients using IVIG or PLEX could mediate a faster improvement of symptoms [11]. In the retrospective review by Safa et al., patients who received IVIG or PLEX as a first-line treatment experienced better MG outcomes than those who received steroids alone (95% vs 63% improvement of MG symptoms, p = 0.011) [11]. Results from the same retrospective study also suggest that IVIG or PLEX may be most effective when used as part of the first-line regimen, as several patients who deteriorated after the initial use of steroids failed to improve despite a second-line use of IVIG or PLEX.

Paradoxical worsening of MG does appear to have occurred in patient 2 of our series after escalation of steroid dose; the evidence for this is the clear improvement in markers of cardiac and muscle inflammation with paradoxical worsening of bulbar symptoms and respiratory failure. Patient 3 also did not respond clinically to high-dose steroids and continued to deteriorate even after the addition of a second immunosuppressant; he showed improvement in CK and troponin with no commensurate clinical response. Both these patients' deterioration and death appear to have been mostly MG-driven due to respiratory failure. Interestingly, these two patients had the highest MGC scores at presentation. While it's certainly difficult to differentiate the symptoms of MG and myositis, an improvement in markers of muscle injury with concurrent worsening of respiratory failure and muscle weakness does make a compelling case for MG even in the absence of autoantibodies or electrodiagnostic tests. MGC scoring may be a useful clinical tool to differentiate between myositis predominant and MG predominant MMM presentations, which may be useful for anticipating likely deterioration. Further evidence from prospective studies is needed. Higher MGC scores appear to align with bulbar symptoms, respiratory failure and poor outcomes. While it's difficult to generalise from such a small series, these cases further add to the emerging importance of early MG-directed therapies (IVIG, acetylcholinesterase inhibitors and PLEX) in MMM syndrome. Prompt commencement of IVIG and pyridostigmine alongside steroids early on in management may help avoid deterioration and poor outcomes. 

There is currently a lack of affordable and reliable indicators that can accurately predict ICI-induced irAEs [16]. Peripheral blood biomarkers have shown some promise in studies as potential predictors of irAEs in patients on ICIs. The NLR reflects the systemic immune condition and may be used as a predictive marker of irAEs. In one meta-analysis, Zhang et al. suggested that baseline NLR can serve as a valuable tool in predicting irAEs. Although a low baseline NLR was predictive of irAEs, the predictive value varied among different types of irAEs, indicating a need for further subgroup analysis in evaluating its efficacy and specific application to MMM syndrome [17].

Matsukane et al. retrospectively analysed 275 cancer patients treated with anti-PD-1 monotherapy, observing 166 irAEs in 121 patients [18]. Relative NLR trends (fold increase) from the baseline in the patients were observed, with significant elevations noted during irAEs. Interestingly, patients experiencing interstitial pneumonitis showed NLR elevation four weeks prior to initial symptoms and diagnosis. Analysing receiver operating characteristic curves revealed that elevated NLR distinguished subsequent pneumonitis severity with high accuracy (AUC 0.93, sensitivity 88.9%, specificity 88.2%, cut-off 2.37, p = 0.0004). After a severe irAE occurred, two NLR trends were observed. Patients who showed a prompt reduction in elevated NLRs had favourable progression-free survival (hazard ratio 0.32, 95% CI 0.10-1.01, p = 0.0140) and overall survival (hazard ratio 0.23, 95% CI 0.06-0.86, p = 0.0057) compared to the patients who maintained persistently elevated NLRs [18]. Interestingly, three out of four patients in our series had baseline NLRs above this cut-off (2.37), and the two with adverse outcome (death) maintained the highest NLRs (41 and 33) compared to those who survived (3.75 and 18.6). While this is a very interesting finding worth pursuing in prospective studies, it’s important to remember that irAEs and treatment response are two sides of the same immunologic coin. Some studies have shown that irAEs predict good response to ICIs [19]. Taking pre-emptive measures to dampen the immune response could potentially affect treatment efficacy. It then becomes imperative to also strategise ways to decouple ICI toxicity from therapeutic efficacy. Strategies like prophylactic cytokine blockade with anti-IL6 and anti-TNF alpha therapies are a key test of whether toxicity and efficacy can truly be decoupled; the results of these strategies so far look promising in preclinical studies [20]. 

While different predictive and prognostic models are being pursued in clinical studies, the PROPHETIC trial stands out as particularly comprehensive. The trial aims to develop an algorithm that combines protein profile data obtained using a proteomic test (PROphet) with clinical information to predict treatment outcomes, including the occurrence of treatment-related adverse effects for stage 4 non-small cell lung cancers and melanomas on ICIs. This idea of risk prediction and stratification has also been promoted outside of clinical trials. The UK immuno-oncology clinical network (IOCN) now recommends baseline cardiac testing for all patients prior to commencing ICIs. This was communicated in their consensus statement published in November 2024 in collaboration with the British cardio-oncology society (BCOS). All patients should have baseline NTproBNP, troponin T/I and ECG prior to starting treatment. Patients are then risk-stratified based on results. All patients in the elevated risk group should be considered for ECHO and referral to cardiologists.

This case series has some limitations. As treating clinicians, we acknowledge the potential for observational or interpretive bias inherent in retrospective synthesis of clinical cases and are aware of the limitations of our small sample size. While every effort was made to report objective findings, management decisions and clinical impressions may reflect the authors’ clinical judgment in a real-world setting. Our approach does not reject established protocols but illustrates a pragmatic adaptation in resource-limited environments. It also aims to encourage honest discussions regarding the effectiveness of specific tests and the reliability of recommendations.

## Conclusions

MMM overlap syndrome is a complex condition requiring a multi-speciality approach to care. Awareness must be raised among urgent and emergency care clinicians through education to facilitate prompt recognition and early optimal treatment since ICI-related toxicities are projected to increase as their clinical indications expand. While aggressive immunosuppression remains the mainstay of treatment, further research is needed to assess the efficacy and safety of various treatment strategies. Head-to-head comparisons between immunosuppressive agents are needed. Context-specific investigation and management protocols should be developed, especially in low-resource settings to maximise cost-effectiveness and efficiency. A prospective head-to-head comparison between the performance of MG autoantibodies and MGC scores in prognostication would be timely.

There is a pressing need for validated predictive and prognostic biomarkers for irAEs to facilitate better decision making and allow exploration of pre-emptive measures in those identified to be at risk. The NLR may be a pragmatic biomarker for both prediction and prognostication of ICI irAEs, particularly in resource-limited settings where specialised immunological testing may be unavailable. While the NLR trends align with outcomes in our series, sample size limitations preclude definitive conclusions about its predictive utility, multicenter prospective studies are needed for validation.

## References

[1] Tarhini A (2013). Immune-mediated adverse events associated with ipilimumab ctla-4 blockade therapy: the underlying mechanisms and clinical management. Scientifica (Cairo).

[2] Twomey JD, Zhang B (2021). Cancer immunotherapy update: FDA-approved checkpoint inhibitors and companion diagnostics. AAPS J.

[3] Long L, Zhang X, Chen F, Pan Q, Phiphatwatchara P, Zeng Y, Chen H (2018). The promising immune checkpoint LAG-3: from tumor microenvironment to cancer immunotherapy. Genes Cancer.

[4] Nishijima TF, Shachar SS, Nyrop KA, Muss HB (2017). Safety and tolerability of PD-1/PD-L1 inhibitors compared with chemotherapy in patients with advanced cancer: a meta-analysis. Oncologist.

[5] Pathak R, Katel A, Massarelli E, Villaflor VM, Sun V, Salgia R (2021). Immune checkpoint inhibitor-induced myocarditis with myositis/myasthenia gravis overlap syndrome: a systematic review of cases. Oncologist.

[6] Haanen J, Obeid M, Spain L (2022). Management of toxicities from immunotherapy: ESMO Clinical Practice Guideline for diagnosis, treatment and follow-up. Ann Oncol.

[7] Kamat S, Patel J, Brown BR, Vyas A (2022). Adverse events induced by nivolumab plus ipilimumab vs. Nivolumab monotherapy among cancer patients: a systematic review and meta-analysis. Cancer Invest.

[8] Shah D, Young K (2023). Exploring pembrolizumab-induced myocarditis, myositis, and myasthenia gravis: a comprehensive literature review and case presentation on bladder cancer. Cureus.

[9] Sánchez-Camacho A, Torres-Zurita A, Gallego-López L (2025). Management of immune-related myocarditis, myositis and myasthenia gravis (MMM) overlap syndrome: a single institution case series and literature review. Front Immunol.

[10] Kooshkaki O, Derakhshani A, Hosseinkhani N (2020). Combination of ipilimumab and nivolumab in cancers: from clinical practice to ongoing clinical trials. Int J Mol Sci.

[11] Safa H, Johnson DH, Trinh VA (2019). Immune checkpoint inhibitor related myasthenia gravis: single center experience and systematic review of the literature. J Immunother Cancer.

[12] Lipe DN, Qdaisat A, Krishnamani PP (2024). Myocarditis, myositis, and myasthenia gravis overlap syndrome associated with immune checkpoint inhibitors: a systematic review. Diagnostics (Basel).

[13] Malvaso A, Giglio P, Diamanti L (2024). Unravelling the acute, chronic and steroid-refractory management of high-grade neurological immune-related adverse events: a call to action. Brain Sci.

[14] Baptista C, Margarido I, Bizarro R, Branco FP, Faria A (2025). Triple M overlap syndrome: myocarditis, myositis and myasthenia gravis after a single administration of pembrolizumab. Cureus.

[15] Lotan I, Hellmann MA, Wilf-Yarkoni A, Steiner I (2021). Exacerbation of myasthenia gravis following corticosteroid treatment: what is the evidence? A systematic review. J Neurol.

[16] Lu HR, Zhu PF, Deng YY, Chen ZL, Yang L (2024). Predictive value of NLR and PLR for immune-related adverse events: a systematic review and meta-analysis. Clin Transl Oncol.

[17] Zhang W, Tan Y, Li Y, Liu J (2023). Neutrophil to lymphocyte ratio as a predictor for immune-related adverse events in cancer patients treated with immune checkpoint inhibitors: a systematic review and meta-analysis. Front Immunol.

[18] Matsukane R, Watanabe H, Minami H (2021). Continuous monitoring of neutrophils to lymphocytes ratio for estimating the onset, severity, and subsequent prognosis of immune related adverse events. Sci Rep.

[19] Wang D, Chen C, Gu Y (2021). Immune-related adverse events predict the efficacy of immune checkpoint inhibitors in lung cancer patients: a meta-analysis. Front Oncol.

[20] Hailemichael Y, Johnson DH, Abdel-Wahab N (2022). Interleukin-6 blockade abrogates immunotherapy toxicity and promotes tumor immunity. Cancer Cell.

